# Accuracy of COuGH RefluX Score as a Predictor of Gastroesophageal Reflux Disease (GERD) in Mexican Patients with Chronic Laryngopharyngeal Symptoms: A Cross-Sectional Study

**DOI:** 10.3390/diagnostics15050636

**Published:** 2025-03-06

**Authors:** Javier Ivanovychs Carrillo-Rojas, Salvador Zavala-Villegas, Guadalupe Morales-Osorio, Fausto Daniel García-García, Mauricio González-Navarro, Viridiana Montsserrat Mendoza-Martínez, Nallely Bueno-Hernández

**Affiliations:** 1 Gastroenterology Department, Specialty Hospital of the National Medical Center “La Raza”, Mexico City 02990, Mexico; ivancarrillorojas@gmail.com (J.I.C.-R.); salva_dor17v@hotmail.com (S.Z.-V.); dramoralesgastro@gmail.com (G.M.-O.); dr.faustog25@gmail.com (F.D.G.-G.); 2 Otorhinolaryngology Department, National Institute of Rehabilitation “Luis Guillermo Ibarra Ibarra”, Mexico City 14389, Mexico; gonavarr@gmail.com; 3 Proteomics and Metabolomics Laboratory, Research Directorate, General Hospital of Mexico, “Dr. Eduardo Liceaga”, Mexico City 06720, Mexico; viiriidiios@gmail.com

**Keywords:** gastroesophageal reflux disease, esophageal pH monitoring, laryngopharyngeal reflux, chronic cough, diagnosis

## Abstract

**Background/Objectives:** Gastroesophageal reflux disease (GERD) is associated with extraesophageal syndromes that require an objective assessment of abnormal acid exposure to establish the diagnosis. The COuGH RefluX score has been proposed as a diagnostic tool for GERD in patients with chronic laryngopharyngeal symptoms. The aim of the study was to evaluate the diagnostic performance of the COuGH RefluX score in the Mexican population. **Methods:** A cross-sectional study was conducted in patients with chronic laryngopharyngeal symptoms. Patients with cough, globus, sore throat, dysphonia, and/or throat clearing of ≥8 weeks duration, 24 h pH-impedance monitoring (pH-IM), and without objective evidence of GERD (defined as acid exposure time >6%) were included in the study. The COuGH RefluX score tool was applied and stratified as low probability with ≤2.5 points, intermediate probability with 3.0 to 4.5 points, and high probability with ≥5.0 points. The kappa test assessed the concordance between both tests; the area under the curve (AUR), sensitivity (S), specificity (E), positive predictive value (PPV), and negative predictive value (NPV) were calculated for each result. **Results:** 164 patients were included; the prevalence of GERD by pH-IM was 32% vs. 40.3% by COuGH RefluX score, the agreement was weak (κ = 0.34; *p* < 0.001), but the AUR was good (0.720 ± 0.17; *p* < 0.001). A score ≤ 2.5 had S = 49%, E = 88%, PPV = 89%, and NPV = 42% to rule out proven GERD, while a score ≥ 5 had S = 65%, E = 71%, PPV = 52%, and NPV = 82% for proven GERD. **Conclusions:** The COuGH RefluX score has low sensitivity but adequate specificity for GERD diagnosis in Mexican patients with chronic laryngopharyngeal symptoms.

## 1. Introduction

Gastroesophageal reflux disease (GERD) affects 13.9% of the world’s population; it manifests with a broad spectrum of symptoms and generates high healthcare costs [1]. It is widely recognized that, in addition to typical esophageal symptoms such as heartburn and regurgitation, extraesophageal syndromes may occur, which have established associations such as cough, laryngitis, asthma, and dental erosions, or proposed associations such as pharyngitis, sinusitis, idiopathic pulmonary fibrosis, and recurrent otitis media [2]. On the other hand, laryngopharyngeal reflux (LPR) is the primary mechanism responsible for extraesophageal syndromes, with an estimated prevalence between 10% and 71%. However, it can be variable and depends on the risk factors and the instrument with which the diagnosis is defined. There is no gold standard, and although objective tests such as multichannel intraluminal impedance-pH monitoring (MII-pH) or pepsin determination in saliva have been developed, they are still rarely available in our setting. Therefore, the diagnosis of LPR is based on the presence of compatible symptoms and signs using validated scales [3,4].

The Reflux Symptom Index is the most frequently used tool. It is a validated questionnaire in several languages that assesses nine symptoms (hoarseness, throat clearing, excess throat mucus or postnasal drip, dysphagia, coughing after eating or lying down, breathing difficulties, troublesome cough, globus, and heartburn); each of them is rated on a scale of 0 to 5 according to severity. An RSI ≥ 13 is strongly suggestive of LPR; however, some of the symptoms are often nonspecific and can be found in healthy people, while other symptoms, such as sore throat or odynophagia, frequently reported in LPR, are not included in the tool, limiting its diagnostic specificity. In addition, severity is based on a subjective visual analog scale that can be modified by external factors [5].

Another widely used test is the Reflux Finding Score (RFS). This tool is based on findings from laryngoscopy examination (subglottic edema, ventricular obliteration, erythema/hyperemia, vocal fold edema, diffuse laryngeal edema, posterior commissure hypertrophy, granuloma/granulation of tissue, and thick endolaryngeal mucus). However, the signs are not specific, have interobserver variability, can be modified by external factors such as smoking, and may even be found in healthy people [6].

Therapeutic testing with proton pump inhibitors (PPIs) is a common strategy for patients with typical esophageal symptoms that has limited utility in the case of laryngeal symptoms, so it is suggested to use objective diagnostic strategies before starting treatment [7,8]. However, due to the limited availability in different sectors of the population, the COuGH RefluX score has recently been proposed as a tool that evaluates esophageal and extraesophageal symptoms, specifically cough, regurgitation, globus, body mass index (BMI), gender, and the presence of hiatal hernia (HH) confirmed by endoscopy; this score estimates the probability of proven GERD during the approach to patients with chronic laryngopharyngeal symptoms, classifying patients who will require an exhaustive approach and limiting the inappropriate use of invasive therapeutic tools or tests [9]. Therefore, this study aimed to evaluate the diagnostic performance of the COuGH RefluX score for the diagnosis of proven GERD in a Mexican population with chronic laryngopharyngeal symptoms.

## 2. Materials and Methods

### 2.1. Study Design

**Figure 1:**
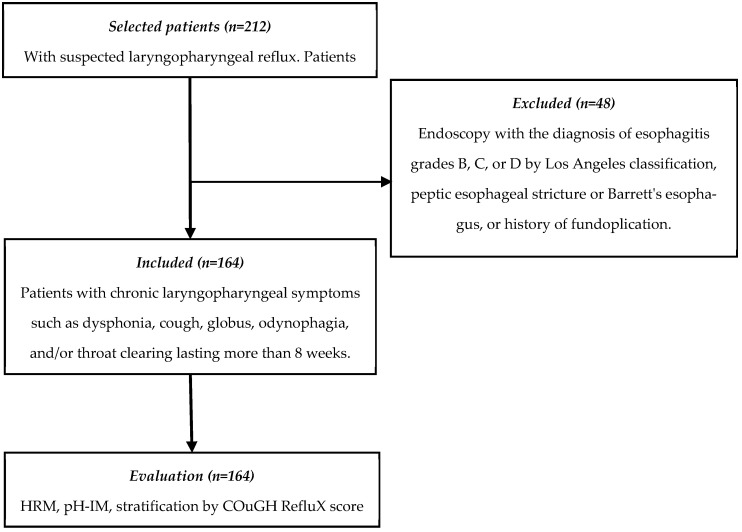
The flowchart illustrates the selection process of the study participants according to the STROBE criteria. HRM: high-resolution esophageal manometry.

A cross-sectional study was conducted based on data from patients retrospectively collected between January 2023 and August 2024, who attended the La Raza Medical Center and General Hospital of Mexico Dr. Eduardo Liceaga (HGMEL). The study was conducted according to the STROBE (Strengthening the Reporting of Observational Studies in Epidemiology) guidelines (Figure 1). All patients had an upper endoscopy, a high-resolution esophageal manometry (HRM), and a pH-IM.

### 2.2. Ethical Considerations

The HGMEL Research Ethics Committee approved the protocol under registration number DI/19/301/03/003. The confidentiality and protection of the participants’ data complied with the legal provisions for protecting personal data, ensuring that the patient will not be identified.

### 2.3. Patient Eligibility Criteria

We included data from patients of both sexes, over 18 years old, with laryngopharyngeal symptoms including dysphonia, cough, globus, odynophagia, and/or throat clearing lasting more than 8 weeks. All patients underwent a screening evaluation with an otorhinolaryngologist and were subsequently referred to the gastrointestinal motility service due to suspected LPR as the cause of the symptoms. The referred patients underwent upper endoscopy, HRM, and pH-IM. Patients with previous evidence of GERD (endoscopy with esophagitis grades B, C, or D of Los Angeles classification, peptic esophageal stricture, or Barrett’s esophagus proven by biopsy) and with a history of fundoplication were excluded.

### 2.4. Clinical, Endoscopic, and Gastrointestinal Physiology Evaluation of Participants

Patient-reported demographic and symptom data of at least 8 weeks duration were collected. Symptoms evaluated included heartburn, regurgitation, cough, dysphonia, throat clearing, odynophagia, and globus. Overweight or obesity was determined by BMI classification (weight in kg/(height in mt)^2^), with overweight being a BMI between 25 and 29.9 kg/m^2^ and obesity being a BMI > 30 kg/m^2^. An upper endoscopy study performed within the previous 6 months was collected to assess the presence of erosive esophagitis (grades B, C, or D of Los Angeles classification) or HH; endoscopic diagnosis of HH was defined by a separation ≥2 cm between the esophagogastric junction (EGJ) and the diaphragmatic clamp.

Previous results of the HRM (ManoScan™ ESO High-Resolution Manometry System, Medtronic, MN, USA) were reviewed to evaluate esophageal motility and to corroborate the type of esophagogastric junction type. The 2 cm separation between the EGJ and the diaphragmatic clamp documented in the reports corroborated the presence of HH [10]. Esophageal motility patterns were reported according to the Chicago classification version 4.0 [11]. Absent contractility was considered if 100% of swallows were failed; ineffective esophageal motility, if ≥70% of swallows were ineffective or ≥50% were failed; if ≥40% of swallows were standard, esophageal motility was considered normal.

Results from the pH-IM (Digitrapper™ Reflux Testing System, Medtronic, MN, USA) were collected; this tool is considered the gold standard. Proven GERD was defined if the acid exposure time (AET) was ≥6%, and unproven GERD if the AET was <6%.

The data collected were used to assess the probability of GERD using the COuGH RefluX score; scores were assigned based on the presence of the following symptoms: cough (1.5 points), overweight (1.5 points), obesity (2 points), globus (−1 point), HH (2.5 points), regurgitation (1.5 points), and male gender (1.5 points). The scores obtained are classified into three categories: low probability if ≤2.5 points, intermediate probability if 3 to 4.5 points, and high probability if ≥5 points.

In addition, a post hoc analysis was performed, excluding the endoscopic finding of hiatal hernia. The COuGH RefluX score was interpreted as low probability (≤1.5 points), intermediate probability (2.0 to 4.0 points), and high probability (≥4.5 points).

### 2.5. Sample Size

For the calculation of sample size, a formula for diagnostic tests was used, where a sensitivity (S) of 71% and specificity (S) of 81% were considered according to the study by Krause et al. [10], with a confidence level of 95% and a power of 80%, obtaining a total of 145 patients. Due to data availability, the final sample size was 164 patients.

### 2.6. Statistical Analysis

Patients were classified into two groups according to their pH-IM diagnosis of GERD. The Kolmogorov–Smirnov test was used to determine the distribution of the variables. Quantitative variables were reported with measures of central tendency and dispersion. The χ^2^ test, Student’s *t*-test, or Mann–Whitney U test were used to see differences between groups as appropriate.

A multivariate logistic regression analysis was performed to check the association of possible risk factors with the presence of proven GERD. The concordance between the diagnostic tests (endoscopy vs. COuGH RefluX score) was evaluated using Cohen’s kappa index (κ); the area under the curve (AUR), S, E, positive predictive value (PPV), and negative predictive value (NPV) of the new diagnostic test were calculated. The statistical analysis was performed with the SPSS V25 statistical package (IBM, Armonk, NY, USA), and a *p*-value < 0.05 was considered statistically significant.

## 3. Results

### 3.1. Demographic Characteristics of the Population

A total of 164 patients were included, of whom 59 (36%) were men and 105 (64%) women; the median age was 54 years (IQR 20.5); 29% had hypertension, 20% had type 2 diabetes (T2D), and 21% were active smokers. The median BMI was 26.1 (IQR 5.9); 75% of patients were overweight or obese. The prevalence of HH was 52% assessed by endoscopy and 25% established by HRM.

Of the included patients, 75% (*n* = 123) reported esophageal and laryngopharyngeal symptoms, and 25% (*n* = 41) reported isolated laryngopharyngeal symptoms. In both groups, chronic cough was the most frequent laryngopharyngeal symptom (69% vs. 80%; *p* = 0.160). Globus was more common in the group with isolated laryngopharyngeal symptoms (44% vs. 24%, *p* = 0.013), while throat clearing was more frequent in the group with esophageal and laryngopharyngeal symptoms (39% vs. 22%, *p* = 0.047) (Table 1).

### 3.2. GERD Diagnosis and Risk Factors

The prevalence of proven GERD was 32% (*n =* 52), higher in the group with esophageal symptoms than patients who reported isolated laryngopharyngeal symptoms (37% vs. 13%, *p* = 0.020). The presence of overweight and obesity was higher in the group with proven GERD (79% vs. 53%, *p* = 0.001) (Table 2). In the multivariate analysis, BMI ≥ 25 increased the risk of GERD (OR 2.72, 95% CI 1.227–6.060, *p* = 0.014); gender, T2D, hypertension, smoking, or the presence of hiatal hernia were not associated with increased risk of GERD.

### 3.3. Diagnosis of Proven GERD According to the Reported Laryngopharyngeal Symptoms

**Figure 2:**
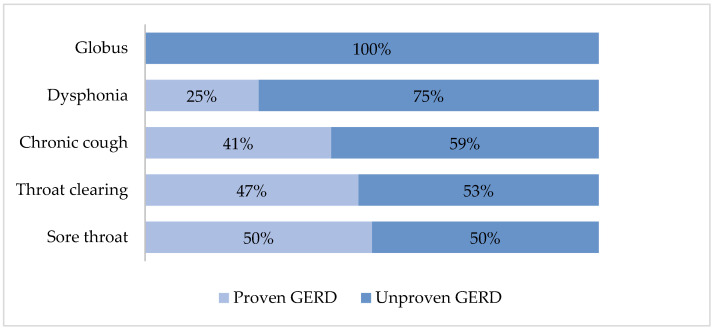
Frequency of GERD according to reported chronic laryngopharyngeal symptoms.

GERD was documented in 31% of patients with chronic cough, 30% of those reporting throat clearing, 23% of the dysphonia population, 15% of the globus group, and 13% of the odynophagia group. A sub-analysis was performed with 63 patients (45%) with only one extraesophageal GERD symptom. Patients with odynophagia (50%) and pharyngeal clearing (47%) had a higher prevalence of GERD (Figure 2).

### 3.4. Proven GERD Prediction Using Cough Reflux Score

**Figure 3:**
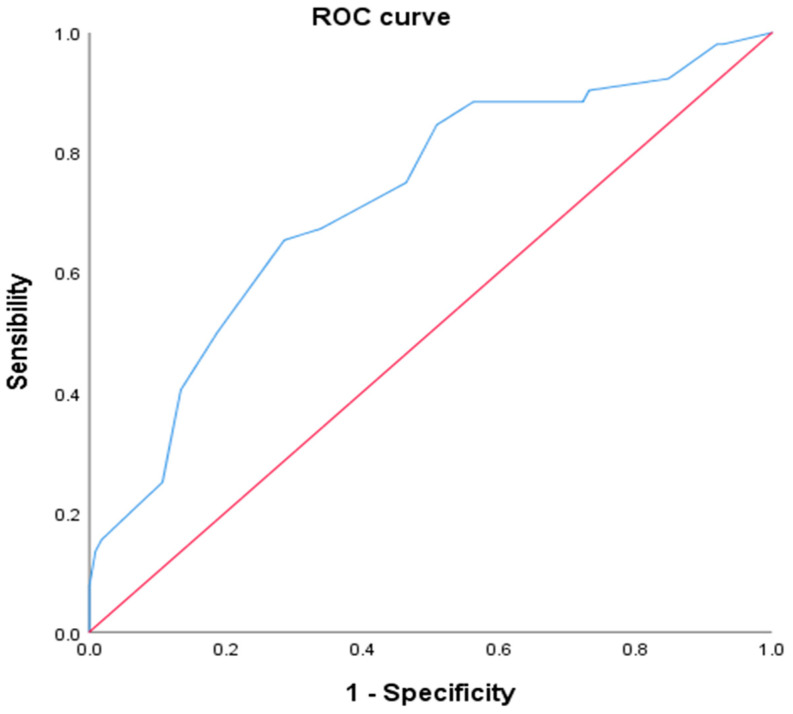
ROC curve for the prediction of GERD using the COuGH RefluX score.

The probability of proven GERD was low in 33.5% (*n =* 55), intermediate in 26.2% (*n =* 42), and high in 40.3% (*n =* 66); by pH-IM, GERD was confirmed in 32% with a statistically significant kappa index (κ = 0.343, *p* < 0.001). The AUR of COuGH RefluX score was 0.720 (95% CI 0.635–0.805, *p* < 0.001) (Figure 3).

**Figure 4:**
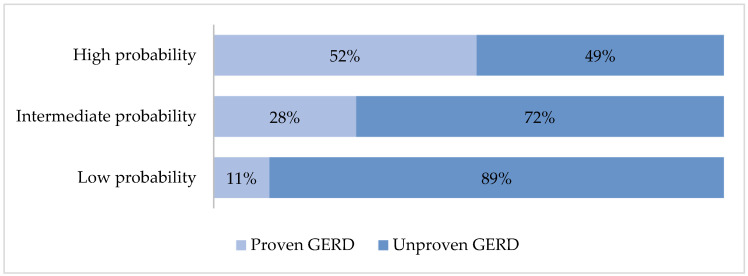
According to the COuGH RefluX score, the frequency of proven GERD in patients with low, intermediate, or high probability.

The prevalence of proven GERD was 51.5% in the high-probability group, 27.9% in the intermediate probability group, and 11.5% in the low-probability group (Figure 4).

A score ≤ 2.5 had an S = 49%, E = 88%, PPV of 89%, and NPV of 42% for ruling out proven GERD, whereas a score ≥ 5 had an S = 65%, E = 71%, PPV of 52%, and NPV of 82% for proven GERD.

### 3.5. Post Hoc Analysis Excluding Hiatal Hernia

The AUR was 0.689 (95% CI 0.602–0.775, *p* < 0.001). The prevalence of GERD was 21%, with a kappa value of 0.120 (*p* = 0.110) when compared with endoscopy. A score ≥ 4.5 had an S = 28.8%, E = 82.1%, PPV of 43%, and NPV of 71% to establish a diagnosis of proven GERD; moreover, a score ≤ 1.5 points had an S = 29%, E = 88%, PPV of 84%, and NPV of 37% to rule out a diagnosis of proven GERD.

## 4. Discussion

Numerous extraesophageal conditions, such as chronic cough, throat clearing, dysphonia, or asthma, have been attributed to GERD; they are estimated to occur in one out of three patients with typical esophageal symptoms. However, they may occur in isolation [12]. Two theories explain the appearance of extraesophageal syndromes in GERD. The “reflux theory” postulates that the loss of the physiological barrier against gastroduodenal contents due to factors such as dysfunction of the upper and/or lower esophageal sphincters, decreased mechanical clearance due to an underlying esophageal motor disorder, or the loss of epithelial tight junctions causes some substances such as hydrochloric acid, pepsin, and bile salts to reach proximal structures such as the pharynx or larynx, leading to epithelial damage, inflammation, ciliary dysfunction and, finally, mucus accumulation, which could explain the presence of dysphonia, throat clearance, or chronic cough. The “reflex theory” proposes that acid exposure in the distal esophagus induces laryngeal symptoms through an indirect mechanism mediated by the vagus nerve, which generates neurogenic inflammation in the airways and produces hypersensitivity to the cough reflex [13,14,15].

Endoscopy is usually the first diagnostic study performed in the presence of heartburn and/or regurgitation; however, it is not recommended in patients with isolated laryngopharyngeal manifestations given the low probability of underlying GERD, and documenting erosive esophagitis is not a sufficient criterion to attribute reflux as the cause of the symptoms [12,16]. Tests such as the gastroesophageal reflux disease questionnaire (GERDQ) have shown low screening capacity to distinguish GERD among patients with chronic cough and are therefore not recommended either [17]. Laryngoscopy is usually the first study performed for those with predominant laryngeal and pharyngeal symptoms. However, it lacks specificity and high interobserver variability, so a diagnosis cannot be established accurately [18,19].

Due to the limitations of conventional instruments, definitive diagnosis requires objective documentation of pathological reflux by pH-IM. However, it remains an expensive and poorly available tool [7,20]. The COuGH RefluX score has been designed to guide therapeutic decision-making, which stratifies the probability of GERD in the evaluation of patients with chronic laryngopharyngeal symptoms.

In our study, a score ≥ 5 points had an S = 65% and E = 71% for proven GERD. Although the test has some limitations, its usefulness is supported by the variables included in the instrument.

First, it has been described that in overweight or obese patients, the pressure gradient between the thorax and the abdomen increases, favoring the return of gastric acid into the esophagus and, therefore, the probability of developing GERD [21,22]. Lechien et al. evaluated a population of 265 patients using MII-pH, finding that patients with a higher BMI had a higher prevalence of GERD and a higher number of reflux events [23]. This observation was corroborated in our population, where overweight/obesity increased the risk of developing GERD threefold.

Another widely recognized risk factor is the loss of the antireflux barrier due to a hiatal hernia. Between 50 and 90% of patients with GERD have a concomitant hiatal hernia; however, most patients with hiatal hernia are asymptomatic [24,25]. In our study, although hiatal hernia was more common in patients with GERD, it was not associated with increased risk in multivariate analysis.

Several symptoms are related to the presence of LPR, but the exact prevalence of each of them is not known. LPR has been reported in 14.2% of patients with chronic cough [26]. LPR has been reported in 14.2% of patients with chronic cough, 10.5% with dysphonia, and 3.5% with pharyngeal clearance [27]. Although chronic cough was the main laryngopharyngeal symptom reported in our study, proven GERD was higher in those with sore throat or throat clearing. A relationship between globus and GERD has been proposed, but the data are inconclusive. The frequent association with typical esophageal symptoms or response to acid suppressive therapy supports causality; however, increased acid exposure time or an increased number of reflux episodes have not been demonstrated and, in the absence of randomized clinical trials, a reliable link between the two entities has not been established [28,29,30]. In both the validation study and our population, globus was a negative predictor of GERD, suggesting the involvement of mechanisms other than reflux.

Finally, other risk factors have also been identified in addition to the variables included in the scale. It has been suggested that smoking increases the risk of developing extraesophageal syndromes, but these findings were not demonstrated in our study [31]. Decreased acid clearance due to esophageal motility disorders has also been linked to GERD. Sykes et al. evaluated 441 patients with chronic cough, asthma, and/or interstitial lung disease, reporting esophageal motility disorders in 66% of the patients [32]. In contrast to these results, in our population, only 21% presented some esophageal motility disorder, and there were no statistically significant differences between patients with and without GERD.

Because LPR is a common cause of chronic laryngopharyngeal symptoms, it is necessary to have tools that allow the stratification of GERD risk and, thus, to adequately select patients who will benefit from using PPIs or diagnostic tests such as pH-IM. This is the first study in a Mexican population that seeks to validate the usefulness of the COuGH RefluX score for predicting pathological reflux in patients with chronic laryngopharyngeal symptoms. We found a low sensitivity but an adequate specificity for a score ≥ 5 points, so the test is helpful in the selection of patients who benefit from ambulatory reflux monitoring. The main limitation of our study is that we do not have MII-pH, which represents the most accurate tool for the diagnosis of LPR. However, the results of our study may be the preamble for new research, with a prospective design, a more significant number of patients that allow for supporting the usefulness of COuGH RefluX score in Mexico, or the design of new scales that assess other laryngopharyngeal symptoms in addition to chronic cough and that do not consider the presence of hiatal hernia, which would facilitate their application in environments with limited resources, where endoscopy is not available.

## 5. Conclusions

The COuGH RefluX score is an accessible test with adequate specificity that can be used in the Mexican population to guide the diagnostic approach in patients with chronic laryngopharyngeal symptoms. Performing pH-IM in patients with ≥5 points to corroborate pathological reflux could be a good diagnostic strategy. Meanwhile, in the population with an intermediate score (3.0 to 4.5 points) or low score (≤2.5 points), a thorough evaluation of alternative causes before considering the diagnosis of GERD could be the best diagnostic strategy, as shown in the graphical abstract.

## Figures and Tables

**Table 1:** Clinical and demographic characteristics of the population.

	Overall *n =* 164	Esophageal and Laryngopharyngeal Symptoms *n =* 123	Isolated Laryngopharyngeal Symptoms *n =* 41	*p*-Value
Age, years (IQR)	54 (20.5)	56 (23)	53 (21)	0.486
Gender, *n* (%)				0.925
Men	59 (36)	44 (36)	15 (37)	
Women	105 (64)	79 (64)	26 (63)	
T2D, *n* (%)	32 (20)	23 (19)	9 (22)	0.649
Hypertension, *n* (%)	47 (29)	39 (32)	8 (19)	0.135
Smoking, *n* (%)	35 (21)	28 (23)	7 (17)	0.441
BMI, kg/m^2^ (mean ± SD)	26.1 (5.9)	26.2 (6.6)	25.7 (5.1)	0.001 *
Overweight or obesity, *n* (%)	123 (75)	77 (63)	23 (56)	0.460
Hiatal hernia on endoscopy, *n* (%)	86 (52)	75 (61)	11 (27)	<0.001 *
Hiatal hernia on HRM, *n* (%)	41 (25)	38 (31)	3 (8)	0.009 *
Esophageal motility pattern, *n* (%)				0.466
Normal esophageal motility	126 (77)	92 (75)	34 (83)	
IEM	35 (21)	29 (24)	6 (15)	
Absent contractility	3 (2)	2 (1)	1 (2)	
Laryngopharyngeal symptoms, *n* (%)				
Chronic cough	118 (72)	85 (69)	33 (80)	0.160
Globus	47 (29)	29 (24)	18 (44)	0.013 *
Throat clearing	57 (35)	48 (39)	9 (22)	0.047 *
Dysphonia	40 (24)	29 (24)	11 (27)	0.675
Sore throat	24 (15)	18 (15)	6 (15)	1.000
Proven GERD	52 (32)	45 (37)	7 (17)	0.020 *

IQR: interquartile range; T2D: type 2 diabetes; BMI: body mass index; HRM: high-resolution manometry; IEM: ineffective esophageal motility; GERD: gastroesophageal reflux. * Significant *p*-value < 0.05.

**Table 2:** Comparison between proven GERD vs. unproven GERD.

	Proven GERD *n* = 52	Unproven GERD *n* = 112	*p*-Value
Age, years (IQR)	56 (23)	53 (21)	0.382
Gender, *n* (%)			0.805
Men	18 (35)	41 (37)	
Women	34 (65)	71 (63)	
T2D, *n* (%)	15 (29)	16 (15)	0.040 *
Hypertension, *n* (%)	17 (33)	30 (27)	0.436
Smoking, *n* (%)	15 (29)	20 (18)	0.110
BMI, kg/m^2^ (mean ± SD)	27.8 (6.2)	25.2 (5.7)	0.002 *
Overweight or obesity, *n* (%)	41 (79)	59 (53)	0.001 *
Hiatal hernia on endoscopy, *n* (%)	35 (67)	51 (45)	0.009 *
Hiatal hernia on HRM, *n* (%)	16 (31)	25 (22)	0.033 *
Esophageal motility pattern, *n* (%)			0.904
Normal esophageal motility	41 (79)	85 (76)	
Ineffective esophageal motility	10 (19)	25 (22)	
Absent contractility	1 (2)	2 (2)	
Laryngopharyngeal symptoms, *n* (%)			
Chronic cough	37 (71)	81 (72)	0.877
Globus	7 (13)	40 (36)	0.003 *
Throat clearing	17 (33)	40 (36)	0.705
Dysphonia	12 (23)	28 (25)	0.790
Sore throat	7 (13)	17 (15)	0.772
Isolated laryngopharyngeal symptoms, *n* (%)	7 (13)	45 (37)	0.020 *

IQR: interquartile range; T2D: type 2 diabetes; BMI: body mass index; HRM: high-resolution manometry; GERD: gastroesophageal reflux. * Significant *p*-value < 0.05.

## Data Availability

We have made the data described in the manuscript publicly and freely available without restriction at https://www.dropbox.com/scl/fi/sogcud6d4nb2f6rg4ndjj/Base-de-datos-para-extenso.xlsx?rlkey=tnjhuq26048l1eqwgqnz4gjsm&dl=0 (accessed on: 20 February 2025).
